# Supplementary material for: Improving needle visibility in LED-based photoacoustic imaging using deep learning with semi-synthetic datasets
Source: Photoacoustics. 2022 Apr 7;26:100351. doi: 10.1016/j.pacs.2022.100351 (PMC9048160; doi:10.1016/j.pacs.2022.100351)
Supplement: MMC S1 — Additional experimental results for model validation and evaluation of impact of needle diameters, input size, and model capacity. [file mmc1.pdf]

Supplementary materials for "Improving needle visibility in LED-based photoacoustic imaging using deep learning with semi-synthetic datasets"

Mengjie Shi, Tianrui Zhao, Simeon J. West, Adrien E. Desjardins,
Tom Vercauteren, and Wenfeng Xia

1 Metrics evaluation

The signal-to-noise (SNR) and the modified Hausdorff distance (MHD) were employed for evaluating the model performance on phantom, *ex vivo*, and *in vivo* data. The quantitative results were reported for each representative image and shown in Figure S1, Figure S2, and Figure S3.

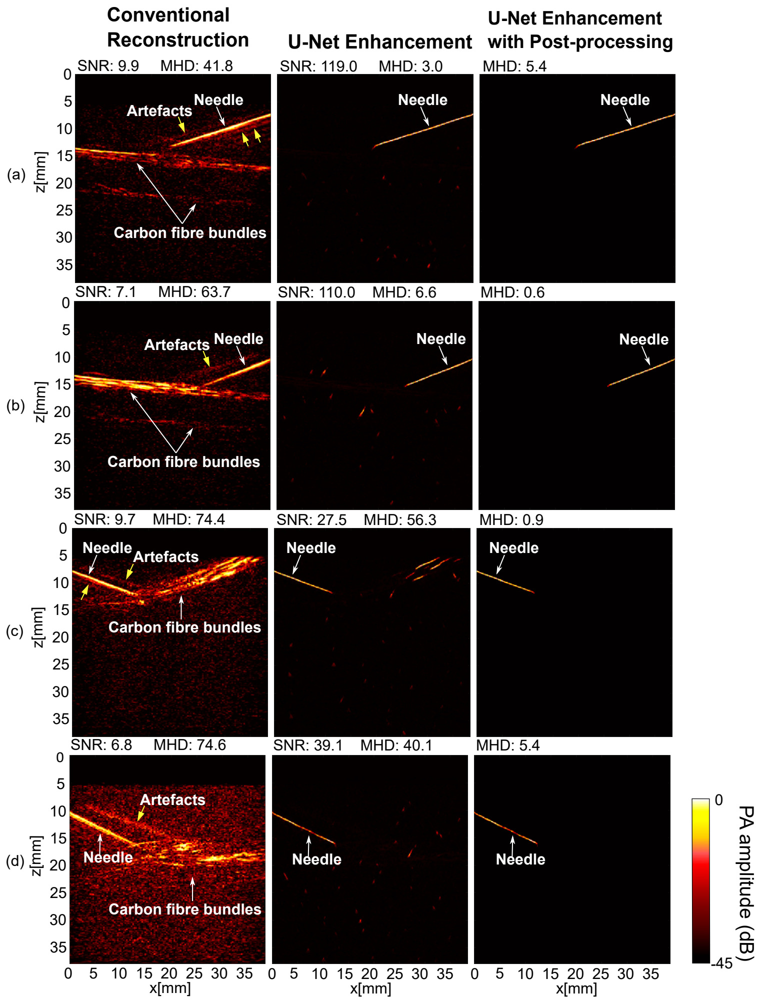

Figure S1: Photoacoustic imaging of needle insertions into blood-vessel-mimicking phantoms with conventional reconstruction, U-Net enhancement, and U-Net enhancement post-processing.

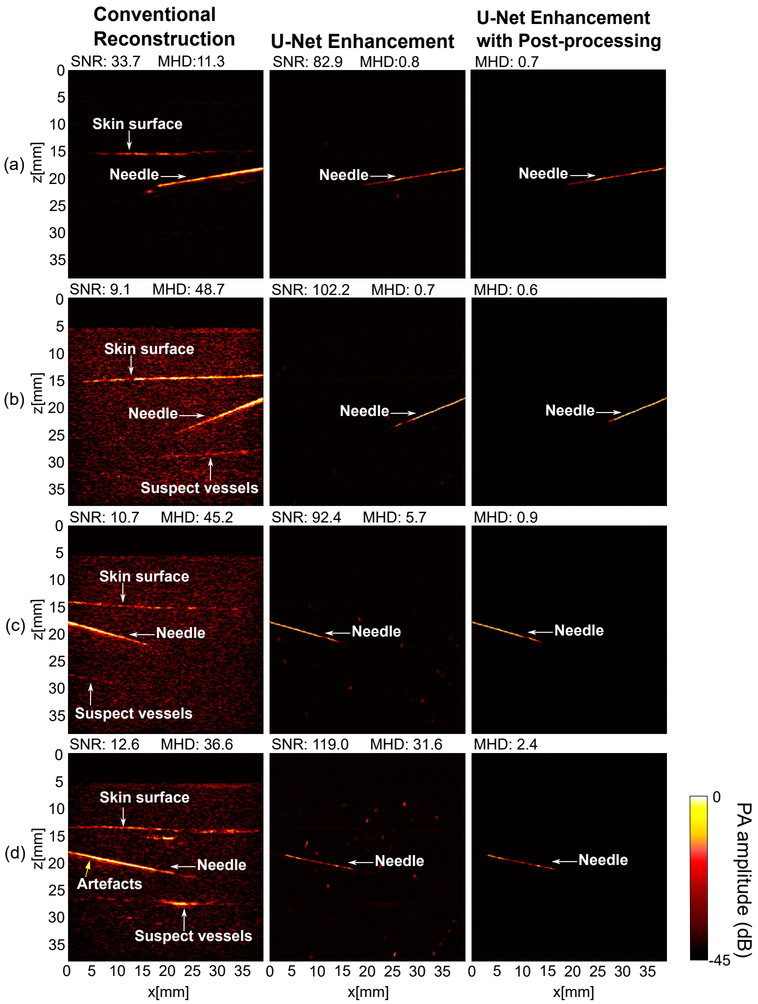

Figure S2: Photoacoustic imaging of needle insertions into pork joint *ex vivo* with conventional reconstruction, U-Net enhancement, and U-Net enhancement post-processing.

2 Impact of number of averaged frames

The relationship between the number of averaged frames (or imaging speed) and the SNRs and MHDs of the needle images with the U-Net enhancement and conventional reconstruction was investigated in Figure S4. The number of averaged frames was increased from 4 to 24 in 2 increments corresponding to an imaging speed decreased from 38 to 6 frames per second if only the data collection time was considered. Four groups of *ex vivo* needle images (20 images for each group) with varying insertion depths and angles were used.

3 Impact of needle diameters

4 Impact of input size

5 Model capacity

Figure S8 demonstrates the learning performance of the U-Net with different scales using the generated semi-synthetic dataset. For all the models, the mean square error (MSE) on the train and validation sets both converged to small values after 5000 iterations. Table S2 and Figure S9 further compare their training

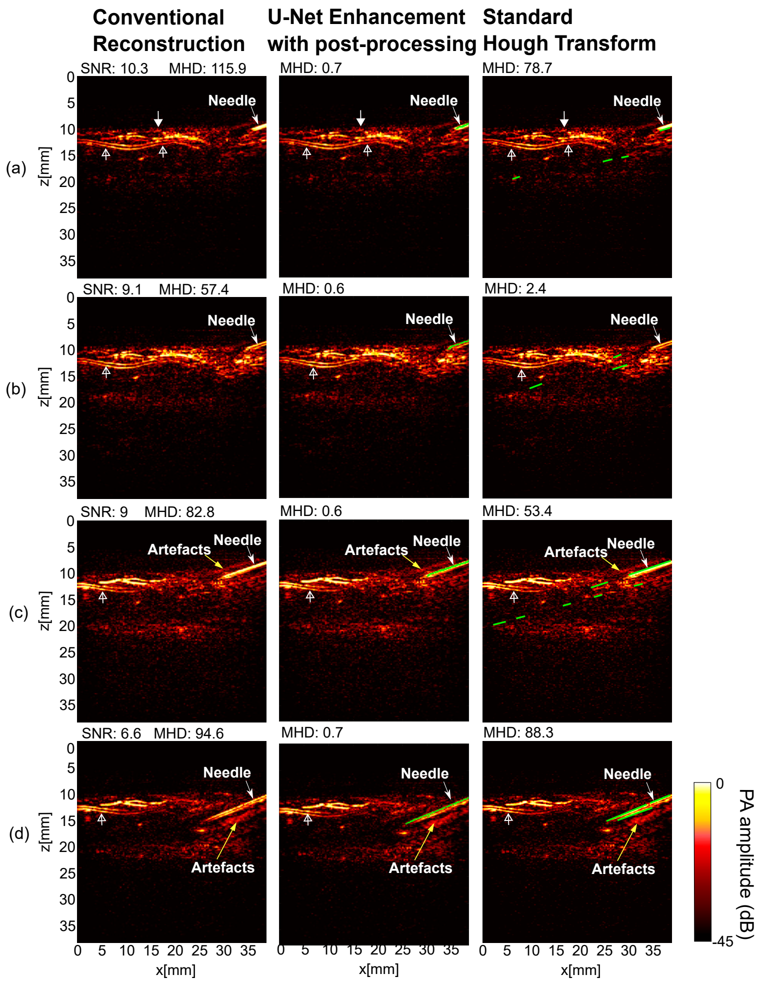

Figure S3: Photoacoustic imaging of needle insertions between two fingers of a human volunteer with conventional reconstruction, U-Net enhancement, and U-Net enhancement post-processing. The outcomes of the U-Net enhancement and the standard Hough Transform (SHT) are denoted by green lines in photoacoustic (PA) overlays. Signals that may correspond to skin and digital arteries are implied by triangle wide arrows and hollow triangle wide arrows respectively.

time, test loss, and inference performance on an *in vivo* PA sequence (128 frames). The proposed model outperformed the models that had larger capacities, indicating its better robustness and generalisability.

Table S1: Quantitative evaluation of the performance of trained neural network with respect to needles with different diameters using measurements on *ex vivo* tissue. The performance metrics are expressed as mean ± standard deviations from 20 measurements acquired at different spatial locations of the *ex vivo* tissue and needle positions.

	SNR		MHD		
	CR	U-Net	CR	U-Net	U-Net with Post-processing
16G	10.9±2.5	106±4.8	51.0±7.3	10.4±5.5	1.1±1.0
18G	9.0±3.1	120.1±9.7	46.4±9.0	6.2±5.9	2.8±3.3
20G	5.7±1.5	81.1±5.2	49.9±13.7	7.6±6.7	4.4±4.6
25G	7.0±3.8	68.9±7.1	45.9±9.6	6.7±7.1	7.8±9.7
30G	9.3±4.3	88.3±5.7	41.1±16.6	6.4±6.0	6.5±6.4

Table S2: Comparison of training and inferring performance between the U-Net model with different scales. The input of the U-Net models has 128 × 128 pixels. The inferred image has 256 × 256 pixels. The test set consisting of 200 images was chosen from the semi-synthetic dataset but blind to the proposed model. The signal-to-noise (SNR) and the modified Hausdorff distance (MHD) were measured on the U-Net enhancement using the inferred data consisting of an *in vivo* sequence of 128 frames.

	5 scales	4 scales	3 scales
Training time (mins)	11.10	12.13	13.43
MSE on test set	45.0±27.4	36.9±18.8	32.5±13.0
Inference time (s)	0.16	0.13	0.09
SNR	95.4±3.9	85.8±14.8	140±35.6
MHD	21.7±29.0	19.8±3.31	4.0±5.9

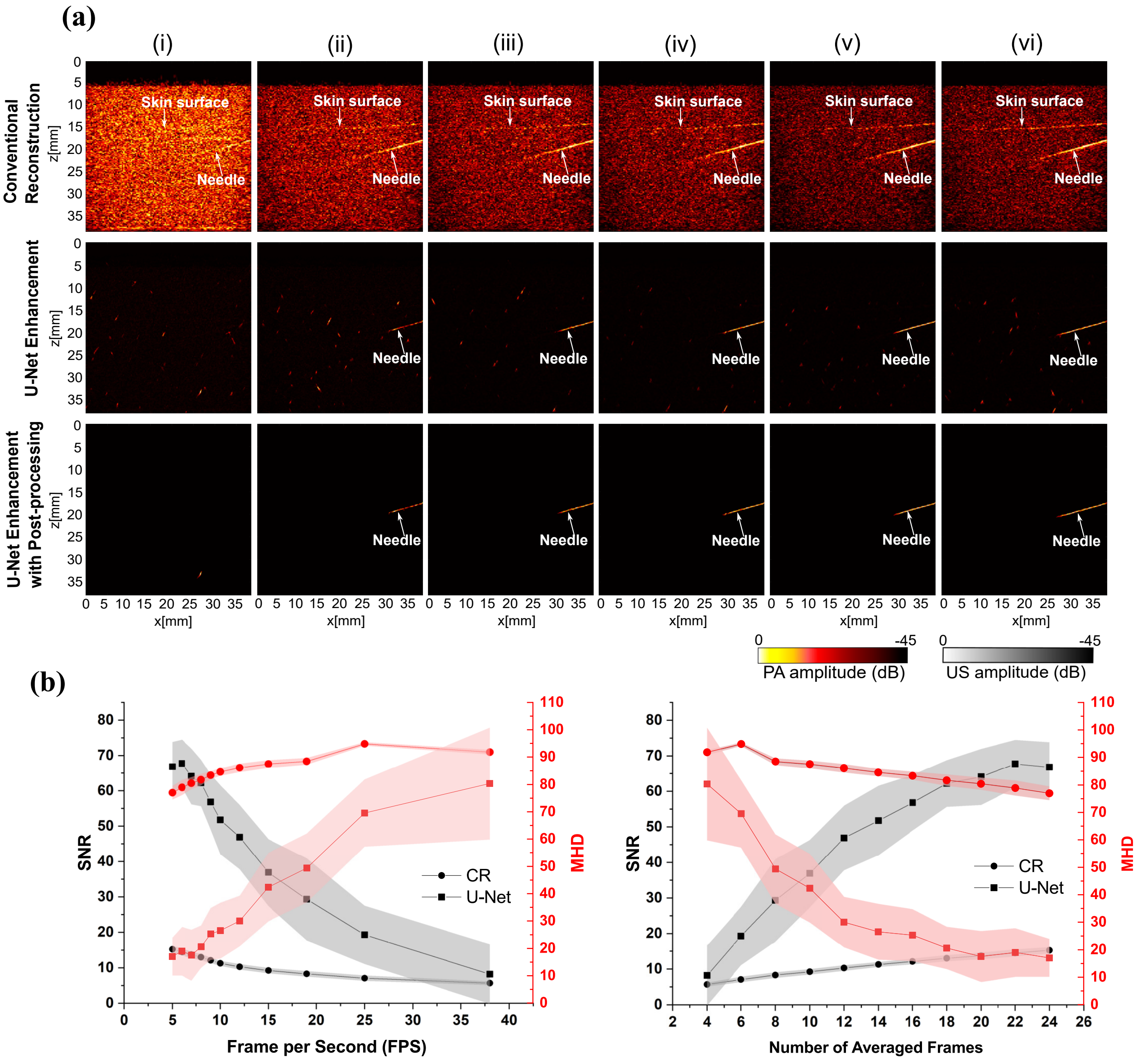

Figure S4: (a) Photoacoustic images of needle insertions into pork joint *ex vivo* tissue with conventional reconstruction, U-Net enhancement and U-Net enhancement with post-processing under different number of averaged frames: from (i) to (vi) are 4, 8, 12, 16, 20, 24 respectively; (b) SNRs of *ex vivo* needle images with conventional reconstruction (CR) and U-Net enhancement under different frames per second (FPS) (left) and different number of averaged frames (right). Data represent average values and shaded error bars represent standard deviations.

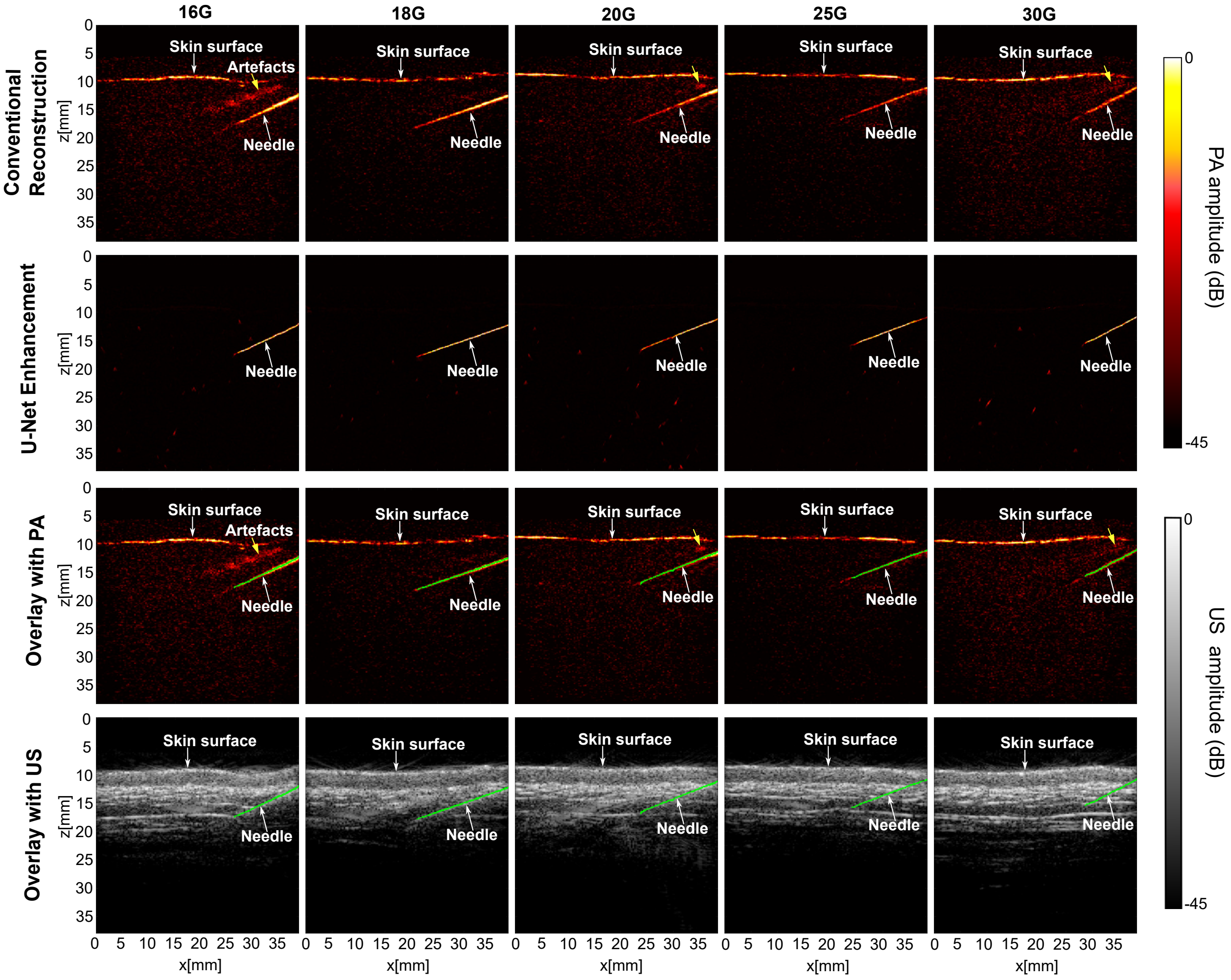

Figure S5: Photoacoustic imaging of needle insertions into pork joint *ex vivo* tissue for needles with different diameters comparing conventional reconstruction, U-Net enhancement, and U-Net enhancement post-processing.

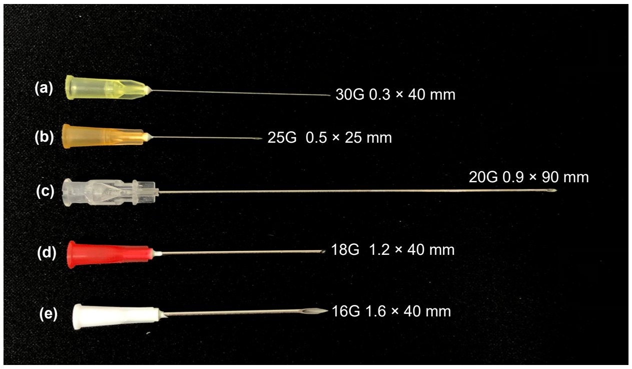

Figure S6: Photograph of metallic needles with different diameters: (a) 16G (b) 18G (c) 20G (d) 25G (e) 30G.

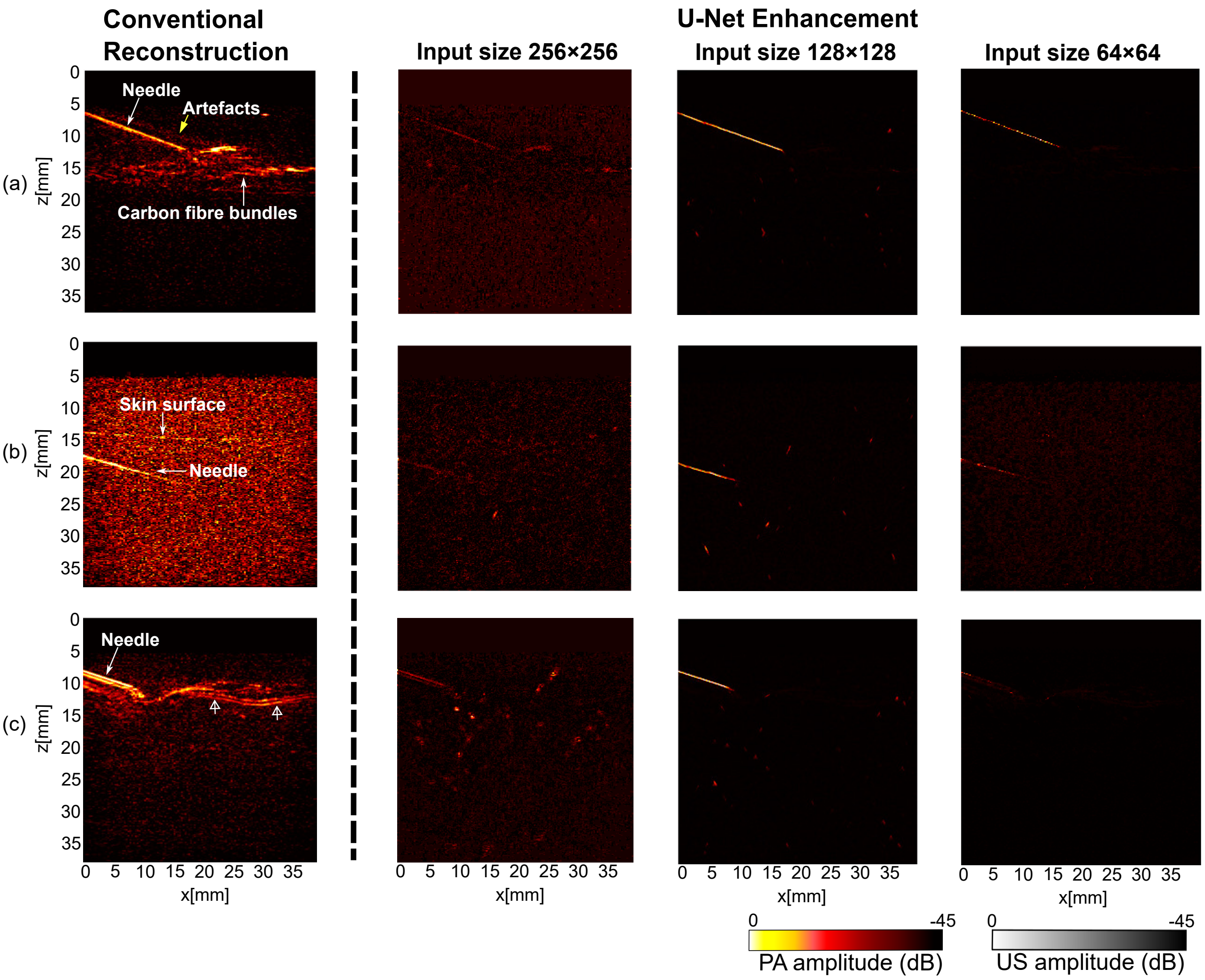

Figure S7: Model inference on different input sizes with real needle images from (a) blood-vessel-mimicking phantoms, (b) pork joint tissue *ex vivo*, and (c) human fingers.

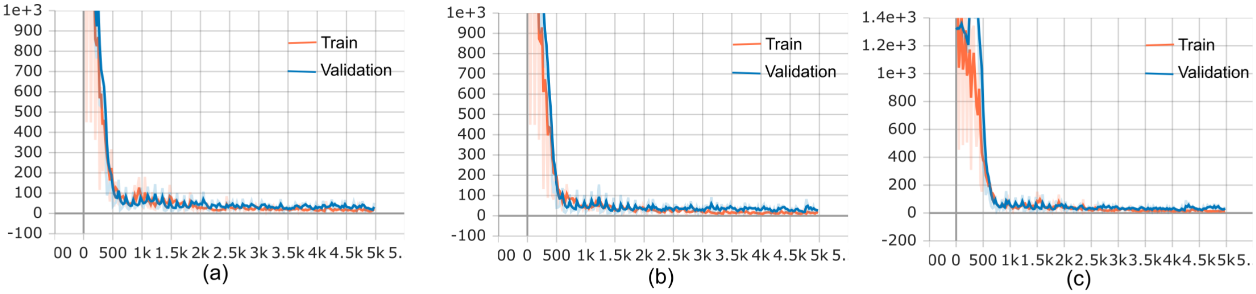

Figure S8: Learning curves of the U-Net with 5 scales (a), 4 scales (b), and 3 scales(c). The generated semi-synthetic dataset, 2000 images with the ground truths, was split into 80% training set, 10% validation set, and 10% testing set. Mean square error (MSE) was reported on the train and validation set per 25 iterations in totaling 5000 iterations.

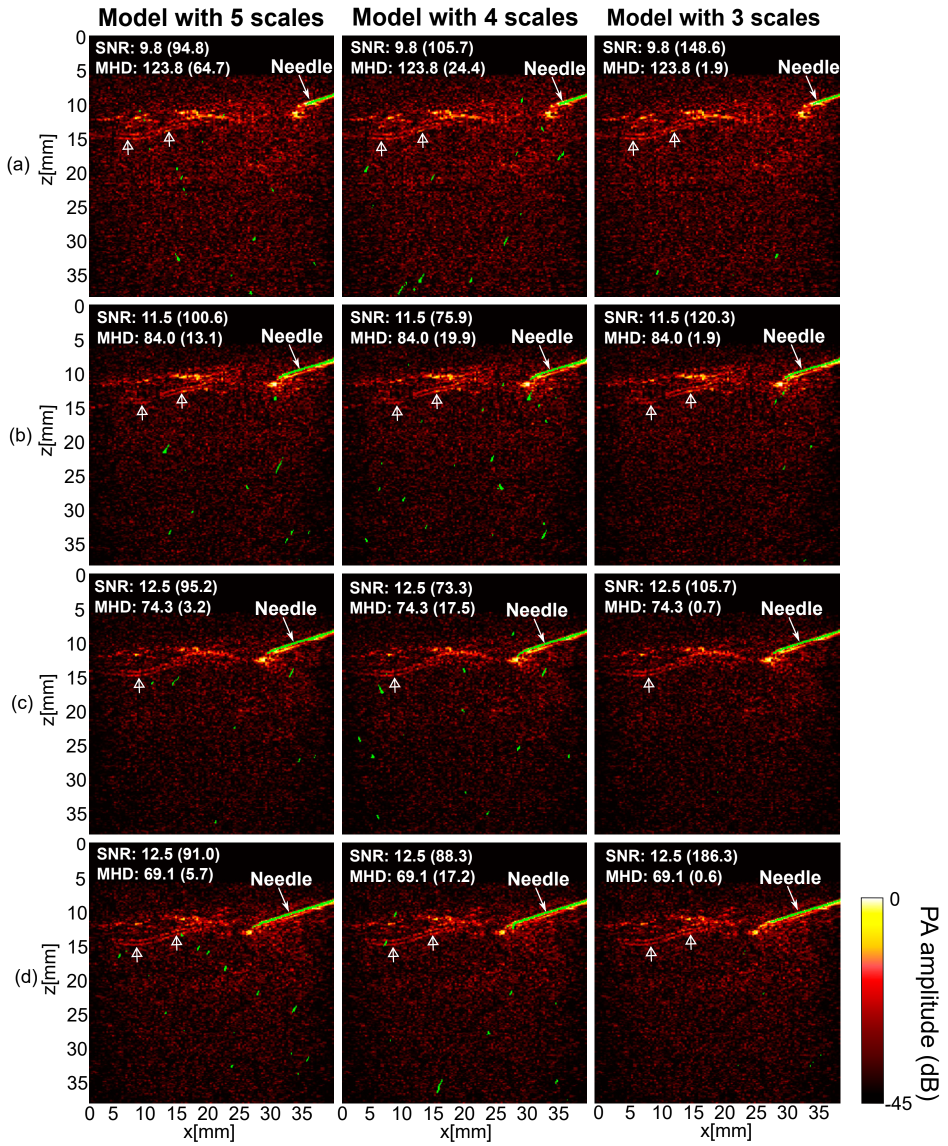

Figure S9: Representative results of model inference on *in vivo* data using the trained model with 5 scales, 4 scales, and 3 scales respectively. The outcomes of the U-Net enhancement are denoted by green lines in photoacoustic (PA) overlays. Signals that may correspond to digital arteries are implied by hollow triangle wide arrows. The signal-to-noise ratio (SNR) and the modified Hausdorff distance (MHD) of the conventional reconstruction (CR) and the U-Net enhancement (in brackets) were measured for each image respectively.
